# Penile Keloid Successfully Cured in a Keloid High-Tendency Patient

**DOI:** 10.7759/cureus.33284

**Published:** 2023-01-02

**Authors:** Izzeddin J Abualjubain, Muath Mamdouh Mahmod Al-Chalabi, Shawaltul Akhma Harun Nor Rashid, Wan Azman Wan Sulaiman

**Affiliations:** 1 Reconstructive Sciences Unit, Universiti Sains Malaysia (USM), Kota Bharu, MYS; 2 Plastic and Reconstructive Surgery, Hospital Raja Perempuan Zainab II, Kota Bharu, MYS

**Keywords:** keloid, keloid treatment, penile keloid, keloid therapy, keloid scar

## Abstract

Keloid scars are a relatively common condition but extremely rare in the penis. This case aims to be added to the previous 34 cases of penile keloid reported in the literature. We present the case of a 15-year-old patient with a high keloid-forming tendency who was successfully cured of penile keloid scarring with excision alone and without any adjuvant therapy. No recurrence was reported over eight years of follow-up. In contrast, at the same time, recurrence is frequently observed in other body parts after excision, making this particular part of the body an area of less keloid occurrence and recurrence.

## Introduction

Circumcision is a simple operation usually performed for various religious, medical, and cultural reasons. It is considered the most frequent surgical procedure performed on males. It is a safe surgery, with a minimal complication rate ranging from 2% to 4%. These complications include bleeding, hematoma, infection, and wound dehiscence that can be seen early postoperatively. Late complications are rarely seen [1]. Penile keloid development is considered a late complication following circumcision, which is very uncommon, with only 34 cases reported in the literature [2], aside from being cosmetically undesirable. However, the penile keloid may affect sexual activity in adult life, and it is socially embarrassing.

Keloids are benign fibroproliferative tumors that form when extracellular matrix (ECM) components, primarily collagen, are deposited abnormally in the dermis and subcutaneous tissue and grow outside the original wound margins [3].

Keloid scars tend to form in patients with dark skin color and positive family history, as well as after trauma, infection, and prolonged wound healing. Particular body areas, such as the ear, shoulder, back, and sternum, are common sites of keloid formation. However, the upper eyelids, palms of the hands, scrotum, and penis are less likely to develop [4,5].

There are a variety of medical and surgical approaches to treat keloid conditions since numerous therapy options have been reported but have either no or minimal benefits.

## Case presentation

**Figure 1:**
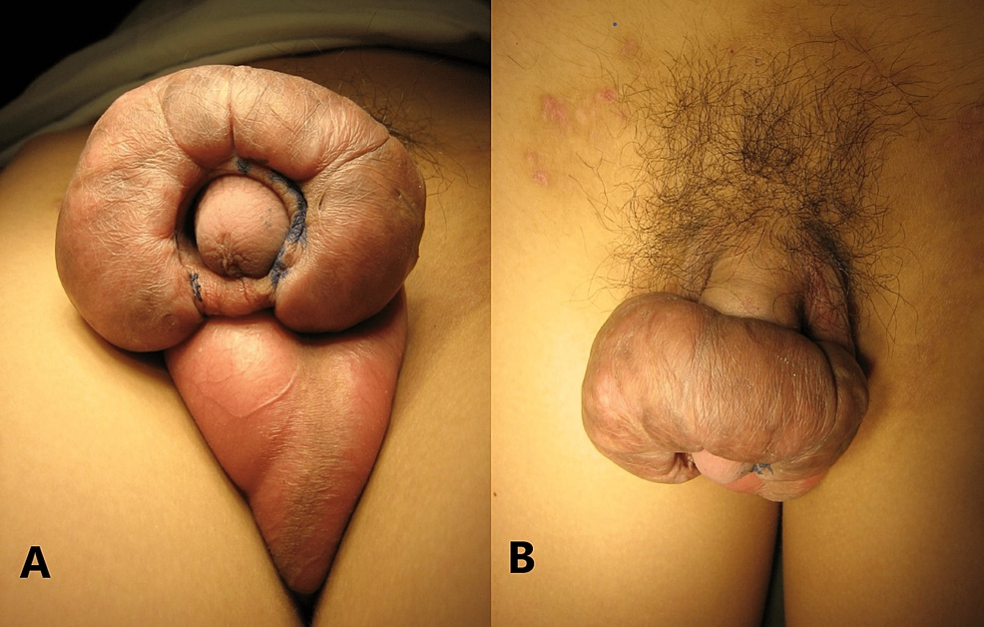
Penile keloid from below (A) and above (B).

A 15-year-old Malay patient presented to our clinic with a significant swelling over the corona of the penis at the site of circumcision for two years, which started to grow slowly six months after ritual circumcision. The patient and his family denied immediate postoperative complications, and the patient had no urological symptoms. On examination, we identified a circumferential, firm mass at the level of the coronal sulcus of the penis, measuring approximately 5 × 4 × 3 cm in its maximum dimensions, as shown in Figure 1.

The decision was made for surgical excision of the mass and local tissue advancement. The operation was conducted under general anesthesia by a plastic surgeon. Two circumferential incisions of 1 mm from the margin encircling the keloid mass were performed. The incisions were deepened to the level of Buck’s fascia, and the mass was dissected off the underlying fascia. Undermining of the surrounding tissue was done, and the penis was partially degloved to allow advancement of the penile shaft skin without tension. The skin margins were sutured with 5-0 Vicryl continuous subcuticular stitches. The excised mass was sent for histopathology, which revealed a large amount of irregular, thick, dense collagen bundles that suggested a keloid. The patient was discharged from the hospital without complications on the first postoperative day. No pre- or postoperative adjuvant therapy was given.

**Figure 2:**
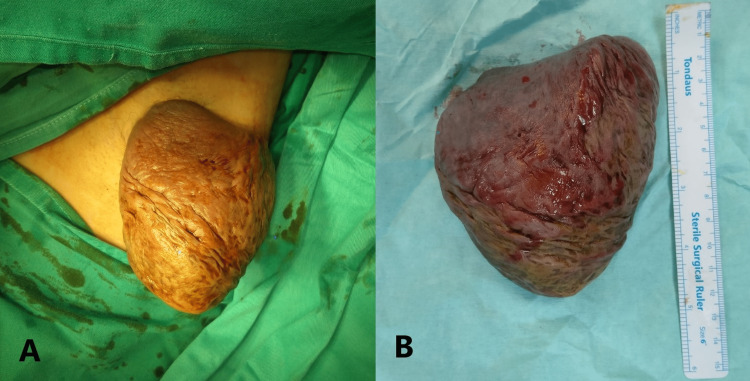
Recurrent left axillary keloid (A) and a keloid specimen (B).

At the same time, the patient also had a large keloid scar over the left axilla, which was excised but reoccurred twice at the same previous surgical site, as shown in Figure 2.

**Figure 3:**
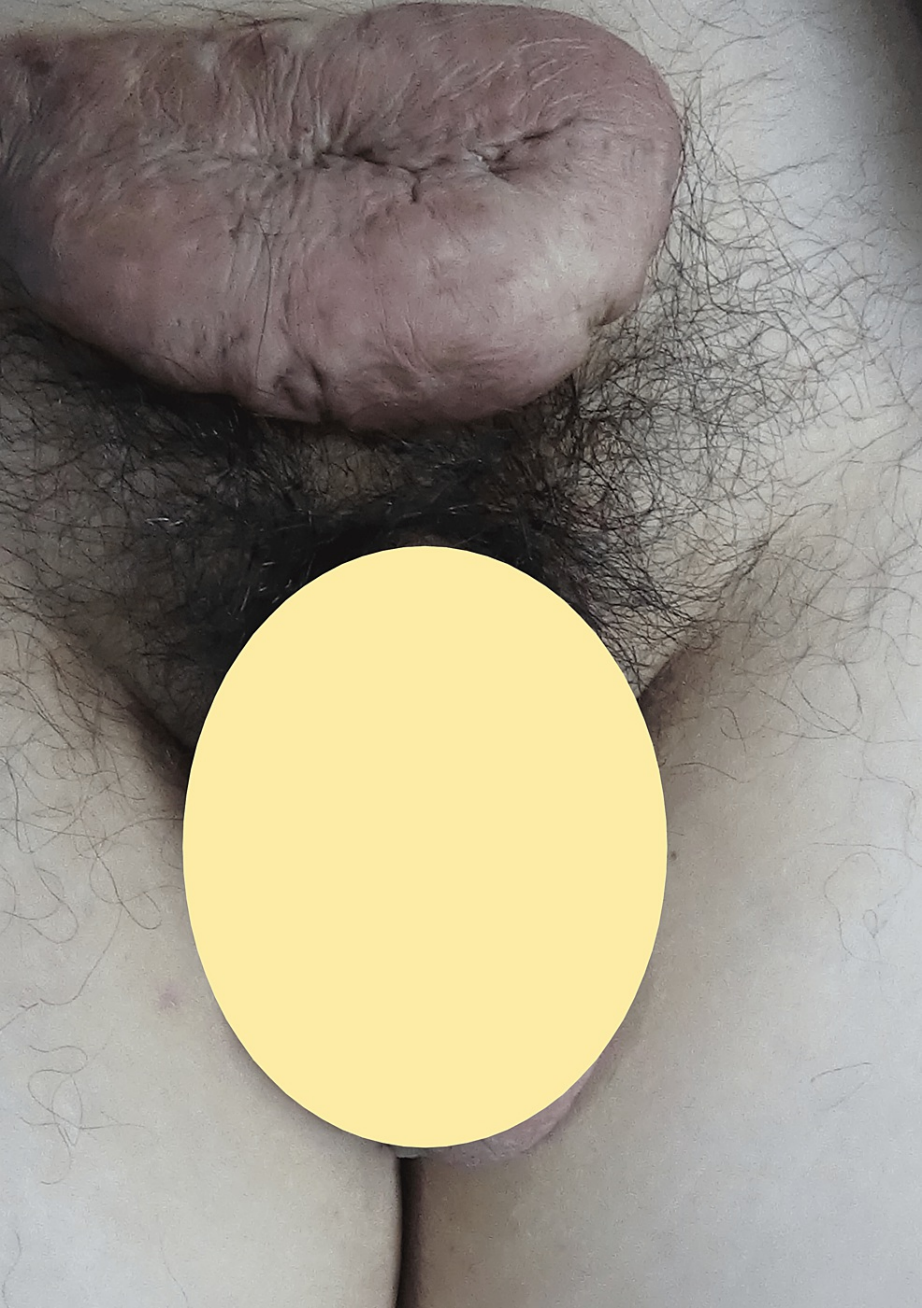
Huge suprapubic keloid.

Additionally, he has keloid scars over the suprapubic region, forearm, thigh, and shoulder, but he is not interested in any treatment due to recurrence (Figure 3).

**Figure 4:**
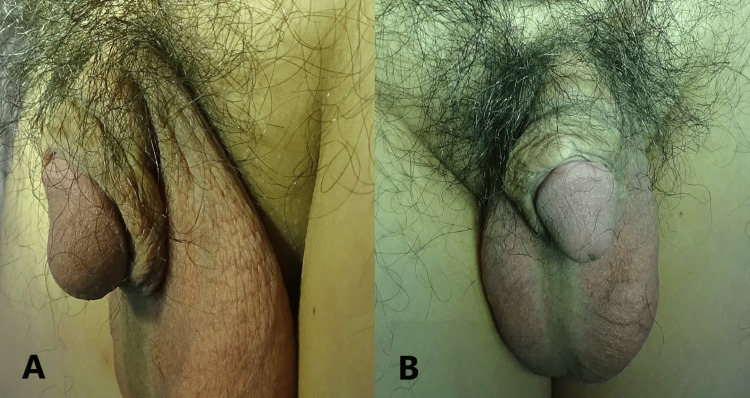
No keloid recurrence after eight-year follow-up: (A) side view and (B) anterior view.

It has now been eight years since the operation. Fortunately, no recurrence was seen over the penile scar, as shown in Figure 4, and the patient is happy with the outcome.

## Discussion

Keloid is a condition with excessive collagen deposition in the dermis and subcutaneous tissue that extends beyond the boundaries of the original wound site. Compared to the linear arrangement of collagen bundles in normal skin or wounds, the bundles are haphazardly arranged, with a predominance of type I collagen over type III collagen fibers, resulting in a high type I to type III collagen ratio [6]. Keloid formation is most common in the ear lobule. However, it can be found in the upper chest, shoulders, upper back, head, and neck and rarely in the penis [7].

Alzeerelhouseini et al. reviewed the causes and treatments for all 34 penile keloid cases published between 1966 and 2021. He discovered that 12 years old is the typical presentation age. The majority of the cases were related to 25 patients’ post-circumcision (73.5%), five patients’ post-surgery (14.5%), three patients’ post-trauma (9%), and one patient’s post-infection (3%). Nine of 29 (31%) patients had a previous history of keloid formation in different areas [2].

The optimal treatment for keloid scars remains undetermined, and no established guidelines are suitable for all patients. The management is empiric, with high recurrence rates overall. Physical and pharmacological therapies are considered nonsurgical treatments for keloids. Examples of physical forms of treatment include pressure therapy and scar massage [8]. Massage therapy is a simple treatment for the patient and family, so it is often recommended as a preventive measure to prevent keloid formation. Still, it has a low level of efficacy when used alone in keloid patients [2]. In our case, pressure therapy was unsuitable because of the scar location. Silicone gel application is another nonsurgical option that is beneficial in softening the scar and preventing its recurrence [4].

Pharmacological management has several forms, but steroids remain the most common agent used either as intralesional injections or in topical forms. The literature showed that significant objective responses to treatment ranged from 31% to 100% and reduced recurrence risk to approximately 50%. However, it may have devastating side effects on the wound-healing process, including ulceration, skin atrophy, dyspigmentation, and telangiectasia [4,8]. General anesthesia exposure may be needed frequently for steroid injections in exceptional cases such as children and in sensitive areas such as the genitalia [4]. Because of the proximity of the keloid lesion to the testes, radiation therapy was not an option in our case. Surgical excision alone is thought to have a high rate of recurrence [8], but it remains the traditional treatment for keloids, especially at the stage when they are causing functional problems. The best treatment is to excise the keloid and minimize the keloid-forming risk factors so that it may need adjuvant therapy pre- or postoperatively. Lee et al. developed the core extirpation technique [9], which requires sufficient removal of actively proliferating and collagen-producing fibroblasts to prevent recurrence with no adjuvant therapy [10]. Some studies have shown promising results with wide surgical excision [11]. Yong et al. reported a case of penile keloid treated by surgical excision with no adjuvant treatments and no recurrence after three years [12].

There are many different treatment modalities for keloids. Some studies showed good outcomes with excision and steroids either pre- or postoperatively [13-16], while others reported successful treatment with excision and silicone gel with scar massage [4,11]. In a meta-analysis of the different keloid treatment options, there were no statistically significant differences between treatment options [11].

In this case, the penile and axillary keloid scars were excised without adjuvant therapy; surprisingly, the recurrence only occurred at the axillary scar after six months, which was excised again with silicone gel application and scar massage adjuvant therapy. In addition, the patient had new keloid scars over the suprapubic region, shoulder, and thigh. Over the past eight years, no recurrence has been observed in the penile scar, raising the question of whether prophylactic measures are necessary to prevent the recurrence of penile keloids, especially in the presence of harmful side effects such as radiation and steroids. It may also conclude that penile keloids tend to recur lower than those forming in other body areas.

## Conclusions

Penile keloid is a rare condition that usually develops following penile surgery. Many different modalities have been proposed to prevent recurrence after excision. In this case, a patient with a high tendency for keloid formation underwent excision for penile and axillary keloids without adjuvant therapy. While the keloid scar recurred shortly after surgery at the axilla site, the penile area had a good outcome with no recurrence. This case showed that penile keloids might have a lower recurrence risk than those forming in other body parts.
